# T-Follicular Regulatory Cells: Potential Therapeutic Targets in Rheumatoid Arthritis

**DOI:** 10.3389/fimmu.2019.02709

**Published:** 2019-11-26

**Authors:** Tingting Ding, Hongqing Niu, Xiangcong Zhao, Chong Gao, Xiaofeng Li, Caihong Wang

**Affiliations:** ^1^Department of Rheumatology, The Second Hospital of Shanxi Medical University, Taiyuan, China; ^2^Pathology, Joint Program in Transfusion Medicine, Brigham and Women's Hospital and Boston Children's Hospital, Harvard Medical School, Boston, MA, United States

**Keywords:** T follicular regulatory cell, rheumatoid arthritis, therapeutic targets, T follicular helper cell, germinal centers, immune regulation

## Abstract

Rheumatoid arthritis (RA) is an incurable aggressive chronic inflammatory joint disease with a worldwide prevalence. High levels of autoantibodies and chronic inflammation may be involved in the pathology. Notably, T follicular regulatory (Tfr) cells are critical mediators of T follicular helper (Tfh) cell generation and antibody production in the germinal center (GC) reaction. Changes in the number and function of Tfr cells may lead to dysregulation of the GC reaction and the production of aberrant autoantibodies. Regulation of the function and number of Tfr cells could be an effective strategy for precisely controlling antibody production, reestablishing immune homeostasis, and thereby improving the outcome of RA. This review summarizes advances in our understanding of the biology and functions of Tfr cells. The involvement of Tfr cells and other immune cell subsets in RA is also discussed. Furthermore, we highlight the potential therapeutic targets related to Tfr cells and restoring the Tfr/Tfh balance via cytokines, microRNAs, the mammalian target of rapamycin (mTOR) signaling pathway, and the gut microbiota, which will facilitate further research on RA and other immune-mediated diseases.

## Introduction

Rheumatoid arthritis (RA) is a systemic autoimmune disease involving damage to the joint synovium and irreversible disability (1). Affecting about five per 1,000 adults, RA threatens the normal work and daily life of patients (2). However, the pathophysiological mechanism of RA is only partly understood. Genotype, environmental factors, and epigenetic modification are implicated in the pathological process of RA (3). Normally, our immune system has extraordinary ability to identify and attack pathogenic microorganisms without targeting self-tissues (4). Genetic defects and environmental factors can lead to deficient immune tolerance, which prevents elimination of autoreactive lymphocytes. This in turn results in abnormal T-cell signaling and autoantibodies, which causes multiple organ inflammation and damage (5). RA is characterized by dysregulated chronic inflammation of the synovial membrane (2, 6). In this inflammatory process, immune homeostasis is disturbed and various abnormal autoantibodies, such as anti-rheumatoid factor (RF) and anti-cyclic citrullinated peptides (anti-CCP), are generated, leading to immune-complex deposition with subsequent destruction of articular cartilage and bone (7, 8).

The generation of autoantibodies related to RA depends on lymphoid follicular germinal centers (GCs). GCs are aggregates of rapidly dividing B cells. In GCs, B cells undergo a series of reactions including affinity maturation, class switch recombination (CSR), and somatic hypermutation, resulting in the generation of large quantities of high-affinity antibodies and memory B cells (9). In this process, T follicular helper (Tfh) cells migrate to GCs and provide the necessary survival, proliferation, and selection signals to cognate B cells (10). Tfh cells are usually characterized as a CD4^+^CXCR5^hi^PD1^hi^BCL6^+^ICOS^hi^ subset in human secondary lymphoid organs (11). The dysregulation of Tfh cells can lead to the production of autoantibodies by B cells. Indeed, the GC reaction requires the regulation and participation of many other cell types, such as monocytes, macrophages, dendritic cells, and neutrophils (12, 13). However, the molecular and cellular mechanisms of autoantibody production are unclear. Traditional therapeutic schedules of RA are based on suppressing excessive immunological responses, which can only achieve stable remission and slow the course rather than cure (12). Moreover, these strategies trigger multiple systemic side effects (2). Therefore, the exploration of novel cellular and/or molecular targets that enable precise regulation of the production of autoantibodies is currently a hot topic.

T follicular regulatory (Tfr) cells, a subpopulation of regulatory T cells (Tregs), have potential for immune regulation within GCs, inhibiting the GC reaction and interacting with Tfh and/or B cells to suppress production of high-affinity antibodies (14–16). Tfr cells are distinguished from other CD4 T-cell subsets predominantly by their Foxp3^+^ and CXCR5 expression (14–16). Dysregulation of Tfr cells leads to an aberrant GC reaction (14, 16), which contributes to the accumulation of autoantibodies and eventually promotes the development of autoimmune diseases (17). Indeed, the Tfr/Tfh ratio and their function are more important determinants of the B-cell response than the absolute number of Tfr or Tfh cells (18). The balance of Tfh and Tfr cells is disrupted in the peripheral blood of patients with autoimmune diseases, such as RA, systemic lupus erythematosus (SLE), myasthenia gravis (MG), and multiple sclerosis (MS) (19–21). The clinical potential of Tfr cells was confirmed by the finding that the frequency of Tfr cells is negatively correlated with the disease activity score in 28 joints based on C-reactive protein (DAS28-CRP) (22, 23). Furthermore, experimental models have highlighted the therapeutic benefit of Tfr cells in mice with arthritis (24). The targeting of regulatory factors and pathways involved in Tfr differentiation and/or function may be a good therapeutic strategy for RA as well as other autoimmune diseases.

## Biology OF TFR Cells

Tfr cells were first described as a subset of Tregs with the surface phenotype of CD4^+^CD25^+^CD69^−^ and a potent suppressive effect in human tonsil GCs (25). Three groups independently discovered specialized Foxp3^+^ Tregs constitutively expressing CXCR5, which can migrate into GCs and regulate the GC reaction (14–16). As CXCR5 is a Tfh-associated molecule and Foxp3^+^ is usually expressed in Tregs, it is generally believed that Tfr cells have characteristics of both Tfh cells and Tregs. Other signaling molecules such as the transcription factors B-cell lymphoma 6 (Bcl-6), cytotoxic T-lymphocyte-associated protein 4 (CTLA-4), inducible T-cell costimulator (ICOS), and programmed cell death 1 (PD-1) are also expressed at different levels in Tfr cells (26). Indeed, Tfr cells have different phenotypes at different differentiation stages or in different locations. For example, mature GC-localized Tfr cells downregulate *Il-2Ra* (CD25) (27). The expression of interleukin (IL)-2 receptor *Il-2Ra* is also decreased in Peyer's patches (PP) Tfr cells, causing them to be unresponsive to IL-2 (28).

Circulating CXCR5^+^ Foxp3^+^ T cells (termed cTfr cells) have been described as the counterparts of tissue Tfr cells (tTfr cells) given that human tissues are unavailable (20, 21, 23). Compared to tTfr cells, little is known about the generation and functions of cTfr cells. cTfr cells are primed by dendritic cells (DCs) and have properties of naive memory cells. They express lower levels of ICOS than lymph node (LN) Tfr cells (29). In one study, cTfr cells even did not express ICOS, PD-1, or Bcl-6 (30). Similar to circulating Tfh, cTfr cells remain for a long time in blood and can be recruited into GCs. In addition, they have weaker suppressive capability than tTfr cells (29, 30). Hence, circulating memory-like Tfr cells are not canonical Tfr cells in terms of function and phenotype. Moreover, the generations of tTfr cells and cTfr cells are also different. The immunized μMT mice (lacking B cells) showed a reduced number of Tfr cells in draining LNs (dLNs) and an unchanged number of blood Tfr cells (29). This indicates that tTfr cells are more likely to develop in a B cell-dependent manner, while cTfr cells are not. Similarly, the frequency of blood Tfr cells is not decreased in B cell-deficient patients (30). It seems that cTfr cells (and cTfh cells) are likely generated when primary Tfr cells leave the LN without passing the B-cell zone, which might lead to incomplete cTfr cell suppression (30). Moreover, both CD28 and ICOS are required for the development of cTfr cells (18, 31). The differences and interplay between tTfr cells and cTfr cells warrant further study.

Tfr cells were initially thought to arise from natural (thymus-derived) Tregs (15, 16). Linterman et al. reported that 97% of Tfr cells express Helios (15). Helios is a transcription factor expressed by thymus-derived Treg cells (32). However, Tfr cells are not found in human thymus (16, 30) but are induced from natural Tregs in the periphery (16). One explanation is that the differentiation of Tfr cells requires multiple stimulations. The microenvironment of the thymus is required for Treg precursor cells to obtain initial molecules such as CD31 and Helios. The differentiation into mature Tfr cells is achieved by subsequent stimulation in peripheral lymphoid tissues (30, 33, 34). Interestingly, in mice, Tfr cells can be derived from naive Foxp3^−^ precursors if adjuvant-promoting T-cell plasticity is used (35).

The differentiation of Tfr cells is a multistep process with various positive and negative regulators (Table 1). Early Tfr cell differentiation may be triggered by antigen presentation by DCs in secondary lymphoid organs (43). The antigen signals initiating Tfr and Tfh cell generation are unclear. Tfr cells differentiate after stimulation by foreign antigens (including ovalbumin and keyhole limpet hemocyanin), self-antigens (myelin oligodendrocyte glycoprotein), or viruses (43). Notably, Tfr cells are more responsive to self-antigens than to foreign antigens (39, 44, 45). This is supported by the fact that Tfr cells prevent a self-reactive B-cell response but do not respond to the influenza-specific B-cell response (39). In addition, Tfr cell counts are higher in insulin (self-antigen)-immunized animals than in ovalbumin (foreign antigen)-immunized animals (45). T-cell receptor (TCR) repertoire analyses have suggested that Tfr and Tfh cells have different TCR repertoires (44). Indeed, the TCR repertoire of Tfr cells may be more similar to Tregs than to Tfh cells, consistent with the similar inhibitory functions of Tfr cells and Tregs (44). Adoption of the canonical phenotype by Tfr cells is likely dependent on interactions with cognate B cells in the GC. In B cell-deficient μMT mice, Tfr and Tfh cell development is abrogated after immunization (15). However, the intracellular signaling events involved in Tfr cell generation are incompletely understood. Notably, the chemokine receptor CXCR5 promotes the migration of Tfr cells into the GC (15, 16). The transcription factors NFAT2 and Bcl-6 upregulate the expression of CXCR5 (15, 16, 36), thereby indirectly promoting Tfr cells differentiation. In addition, Tregs lacking Bcl-6 expression cannot develop to Tfr cells. While loss of Blimp-1, a transcriptional repressor, downregulates Bcl-6 expression, it increases the number of Tfr cells. Given that, Bcl-6 may be required for Tfr cells generation, while Blimp-1 restrains the expansion of Tfr cells (through Bcl-6 inhibition) (15). Moreover, full Tfr differentiation relies on costimulatory molecules (CD28, ICOS), co-inhibitory molecules (PD-1), and cytokines (IL-2, IL-6, and IL-21) (15, 18, 29). Sage et al. (18) found that Tfr counts are very low in both LNs and blood of mice lacking CD28 or ICOS. CD28 may mediate the interaction with B cells and DCs in the process of Tfr generation because B7 (CD28 receptor) and ICOS ligand (ICOSL) are expressed in B cells and DCs (46). CD28 costimulation is required for Foxp3 expression during Treg differentiation (47). ICOS controls persistent Tfh cell migration into GCs in a manner independent of antigen presentation by DCs or cognate B cells (48). In addition, ICOS activates phosphoinositide-3-kinase (PI3K) signaling, which is crucial for the induction of Bcl-6, MAF, IL-4, and IL-21 (49), and therefore influences Tfh cell differentiation (50–52). These functions of CD28 and ICOS might also occur in Tfr cells.

**Table 1:** Factors involved in Tfr cell differentiation.

**Molecules**	**Regulatory mechanism**	**Possible effect**	**References**
**Transcription factors**			
Bcl-6	Promotes the expression of CXCR5	Enhance	(16)
Blimp1	Downregulates the expression of CXCR5	Inhibit	(15, 16)
NFAT2	Promotes the expression of CXCR5	Enhance	(36)
FOXO1	Inhibits ICOS expression	Inhibit	(37)
**Costimulatory and coinhibitory signals**			
CD28	Likely maintains FoxP3 expression	Enhance	(18)
ICOS	Mediates expression of Bcl-6	Enhance	(18)
PD-1	Binds to PD-1 ligand	Inhibit	(18)
**Cytokines**			
IL-21	Reduces the expression of CD25 by upregulating Bcl-6 in Tfr cells and thereby lowers responsiveness to IL-2	Inhibit	(24, 38)
IL-2	Upregulates Blimp-1	Inhibit	(39)
IL-6	Downregulates FOXP3 transcription factors; might activate STAT3 pathway	Unknown	(22)
**miRNA**			
miR-17~92	Targets PTEN and enhances PI3K-Akt-mTOR signaling	Enhance	(40)
miR-146a	Might target ICOS and inhibit NF-κB signaling	Inhibit	(41)
miR-99	Represses mTOR signaling	Inhibit	(42)
miR-155	Suppresses SOCS1 (an inhibitor of IL-2R signaling in Treg cells)	Enhance	(42)

Both Tfh cells and Tfr cells show high levels of PD-1 expression (15, 16). The impact of PD-1 in humoral immunity is complex. Although PD-1 suppresses the generation of long-lived plasma cells through reducing Tfh cell-associated cytokine production (53), blockade of PD-1 signaling pathway leads to enhanced humoral immunity (54, 55). Available data support PD-1 as a positive regulator of Tfr cell differentiation. In the lymph nodes of PD-1/PD-L1-deficient mice, the number of Tfr cells is decreased (18). Moreover, PD-1 inhibits differentiation and function of Tfh cells (18, 54). Interestingly, PD-L1 signaling might promote Foxp3^−^ T precursors differentiating into Tfr cells under specific circumstances (35). CTLA-4, which is often expressed in Tfr cells and Tregs, also regulates the differentiation of Tfr and Tfh cells (56, 57). Deletion of CTLA-4 at the start of immunization results in increased numbers of Tfr and Tfh cells (57). In addition, CTLA-4-deleted Tfr cells do not suppress Tfh and B cells (57). The influence of CTLA-4 in Tfr cells seems to be related to IL-10 and/or B7 (57, 58). A recent study suggested that ICOS signaling inactivates the transcription factor FOXO1 to promote Tfh cell differentiation (37). The role of FOXO1 in Tfr cells differentiation is largely unknown but might be related to the regulation of Bcl-6 expression. The expression of STAT3 in Tregs is also important for Tfr cell differentiation in the spleen and in Peyer's patches (PPs). Tfr cell differentiation were severely blocked in Stat3CD4KO mice after immunization (59). Moreover, enhanced IL-6/pSTAT3 signaling might promote Tfh generation and thereby lead to an imbalance in circulating Tfh cells and Tfr cells (22). Indeed, IL-6 is recently proposed to allow GC Tfh cell generation under sustained TCR stimulation without response to inhibitory IL-2 signaling by negatively regulating IL-2Rβ (CD122) expression (60). It is likely that tumor necrosis factor receptor-associated factor 3 (TRAF3) is a particularly crucial mediator of Tfr differentiation (61). In TRAF3^Treg−Ko^ (TRAF3-deficient) mice, the generation of Tfr cells is impaired after SRBC immunization. In addition, TRAF3 regulates ICOS expression via the ERK-AP1 signaling pathway in Tregs (61) and possibly also in Tfr differentiation.

The mammalian target of rapamycin (mTOR) signaling pathway seems to regulate the generation and function of Tfr cells (62). It is reported that the conversion of Tregs into Tfr cells can be precluded when the PI3K-mTOR signaling pathway is inhibited by Roquin (through upregulation of Pten) (40). Furthermore, the development of Tfr cells is regulated by miRNAs such as the miR-17–92 clusters, miR-146a, miR-99, and miR-155 (42). Moreover, the differentiation of Tfr cells is under the control of several cytokines. For example, IL-2 inhibits Tfr development (39) even though it supports the generation and function of Tregs (63). IL-21 suppresses Tfr differentiation through activating STAT3 signaling in BXD2 autoimmune mice (24). This regulation of IL-2 and IL-21 on Tfr generation will be discussed below.

Most of the described studies regarding the biology of Tfr cells were based on mice. This raises the question of whether Tfr biology in mice is replicated in humans. Indeed, there are a few differences between mouse and human Tfr cells. For example, the maintenance of Tfh cells and Tfr cells in LNs does not require GC B cells in patients receiving rituximab (rituximab depletes GC B cells in the LNs) (64). By contrast, in mice, Tfr cells developed in a B cell-dependent manner (15). Moreover, given that Tfr cell differentiation is regulated by various cells, whether the above regulators directly or indirectly (via their effects on other immune cells involved in the process of Tfr differentiation) act in Tfr cells is unclear.

## Functions and Regulatory Mechanisms of TFR Cells

Tfr cells in GCs play an important regulatory role. They suppress humoral immunity by inhibiting Tfh and B cells (14–16). Specifically, they suppress downstream effector responses in B cells, including CSR and somatic hypermutation rather than initial B-cell activation (65). Suppressed B cells show a lower metabolic level in *in vitro* cocultures, even in the absence of Tfr cells, suggesting that Tfr cells regulate B cells at the epigenetic level (65). Notably, this regulatory function requires participation of Tfh cells. Tfr cells do not inhibit B cell proliferation in cocultures of Tfr cells and B cells (without Tfh cells) (26, 65). Whether the suppressive effect on B cells is a direct effect of Tfr cells or a consequence of Tfh cell dysfunction caused by Tfr cells is unclear. Furthermore, Tfr cells inhibit Tfh cell proliferation and the production of IL-4 and IL-21 (29, 65). IL-21 has a widely known function in the generation and maintenance of GCs and is essential in the development of RA (66). Sage et al. (43) also proposed that Tfr cells induce the death of B cells and Tfh cells by secreting granzyme B, as do Tregs.

**Figure 1:**
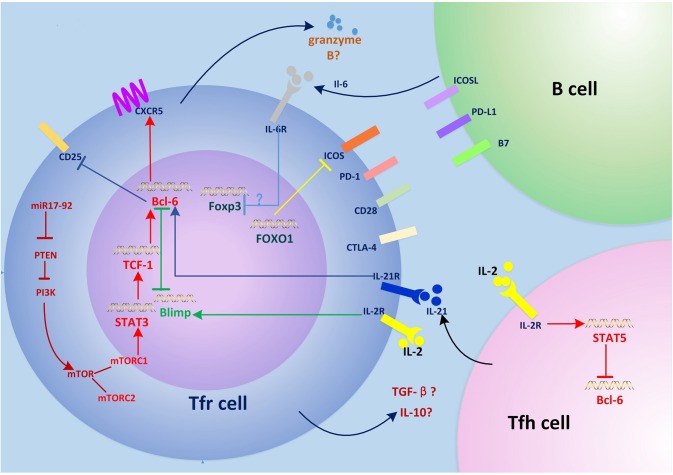
Regulation of Tfr cell differentiation and function. The miR-17–92 clusters enhance PI3K-Akt-mTOR signaling by inhibiting PTEN, which promotes conversion of regulatory T (Treg) cells into T follicular regulatory (Tfr) cells. mTORC1 phosphorylates STAT3 and thereby induces expression of the transcription factor TCF1, which upregulates Bcl-6, inducing surface expression of CXCR5 and the migration of Tfr cells into germinal centers (GCs), where they regulate the GC response. Interleukin (IL)-6 signaling may downregulate FOXP3 and activate the STAT3 pathway at this stage. Interactions between Tfr cells and B cells such as ICOS-ICOSL and CD28-B7 are also required for Tfr cell generation. Tfr cells differentiation is inhibited by PD-1/PD-L1. IL-21 signaling also negatively regulates Tfr cell differentiation by promoting Bcl-6-mediated inhibition of CD25 (IL-2 receptor) and lowering responsiveness to IL-2. FOXO1 negatively regulates Tfr cell differentiation by inhibiting the expression of ICOS. Tfr cells have a suppressive effect on T follicular helper (Tfh) and B cells by secreting granzyme B, transforming growth factor (TGF)-β, and IL-10. The expression of CTLA-4 in Tfr cells inhibits this process.

The regulatory mechanism of Tfr cells depends on multiple factors and pathways (Figure 1), for example, the direct suppressor CTLA-4. Loss of CTLA-4 in Tfr cells results in defective suppression of antigen-specific antibody responses (56, 57). Tfr cells inhibit Tfh cell generation, IL-4 production by Tfh cells, and the expression of CD80 and CD86 on B cells in a CTLA-4-dependent manner, which is crucial for restraining the GC reaction (56). Moreover, transforming growth factor (TGF)-β production by Tfr cells serves as a crucial role in preventing Tfh cell accumulation, self-reactive B-cell activation, and autoantibody production (67). The role of IL-10 in Tfr cell suppression is not fully clarified. IL-10 is a pleiotropic cytokine that provides a survival signal to B cells and regulates antibody production (68). It is likely that Tfr cells inhibit IL-10 production by Tfh cells (29, 69), thereby suppressing aberrant GC responses. By contrast, the level of IL-10 mRNA is high in Tfr cells (15) and Tfr cell product IL-10 to support GC B cell proliferation and GC response in acute infection with lymphocytic choriomeningitis virus (LCMV) (70). IL-10 deficiency in B cells prevented immune tolerance, which results in decreased Tfr cells and increased IL-21 expression by Tfh cells (71). Moreover, Tfr cells express the IL-1 decoy receptor and the IL-1 receptor antagonist to suppress the activation of Tfh cells (72). Notably, the ability of Tregs to suppress the expansion and pathological activity of naive T cells is impaired in TRAF3^Treg−Ko^ mice (61). The mechanism underlying this process and whether a similar phenomenon occurs in TRAF3^Tfrcells−Ko^ mice need to be determined.

## TFR Cells in RA

As in other autoimmune diseases, the development and progression of RA are unclear. The chronic inflammation in RA is associated with multiple immune cells (73). It has been confirmed that Tfr cells regulate the GC reaction in mice (14–16). However, the involvement of Tfr cells and their relationships with other cell subsets in human autoimmune disease, such as RA, are still poorly understood.

Evidence regarding the participation of Tfr cells in RA as well as other autoimmune diseases is conflicting. Niu et al. (22) revealed suppressed differentiation of Tfr cells in patients with RA, accompanied by high levels of PD-1 and IL-21, negative regulators of Tfr cell differentiation. The frequency of circulating Tfr cells is decreased and negatively related to autoantibody [immunoglobulin G (IgG), RF, anti-CCP] levels and disease activity in patients with RA (22, 23). A similar decrease in circulating Tfr cells in active RA was reported by Romão et al. (74). Liu et al. (23) showed that inactive patients with RA have significantly increased circular Tfr cells compared to healthy controls (HCs). Patients with stable-remission RA show increased activation of Tfr subsets along with enhanced inhibition of Tfr cells to Tfh cells (23), suggesting that an increased number of Tfr cells suppress autoimmunity in patients with RA, stabilizing their condition. Importantly, restoration of the number of Tfr cells in mouse models inhibits aberrant immune responses. For example, transferring Tfr cells from BXD2-IL21^−/−^ mice into young BXD2 mice suppresses the GC response and the production of autoantibodies (24). Lee et al. observed an increase in the number of Tfr cells in the spleen of collagen-induced arthritis (CIA) mice treated with intravenous immunoglobulin (IVIG), which was accompanied by a reduced serum level of RF (75). These studies suggest that the reduction of circulating Tfr cells is closely related to the development of RA and that restoration of the number of Tfr cells would ameliorate autoimmune responses. However, the increased number of Tfr cells in RA was also reported (76). Romão et al. (74) also found that inactive patients with RA and HCs have similar frequencies of circulating Tfr cells, which is not in line with Liu et al. In addition, the percentage of circulating Tfr cells in CD4^+^ T cells was significantly reduced in an methotrexate (MTX) treated group compared to a non-treated group, suggesting that therapeutic schedule affects the frequency of Tfr cells. Moreover, similar increases in the number of circulating Tfr cells have been found in patients with other autoimmune diseases such as SLE, new-onset ankylosing spondylitis, and SS (30, 77–79).

Dysregulation of Tfh cells is strongly linked to the pathogenesis of autoimmune diseases (80–82). An increased number of circulating Tfh cells has been reported in patients with RA (23, 76, 83). Moreover, in patients with primary Sjögren's syndrome (SS), activated Tfh cells in peripheral blood correlate with disease activity (78). In one study, the frequencies of circulating CD86^+^CD19^+^ activated B cells and CD3^+^CD4^+^CXCR5^+^ICOS^+^PD-1^+^ Tfh cells in patients with new-onset RA were higher than those in HCs and were positively correlated with disease activity (83). After treatment with disease-modifying antirheumatic drugs (DMARDs) and a Chinese herb, the frequencies were reduced significantly (83). The increase in circulating Tfh cells also occurs in patients with active or inactive RA (23). However, Romão et al. (74) found that the frequency of Tfh cells in patients with inactive RA is less than that in HCs possibly due to the heterogeneity of patients.

The Tfr/Tfh ratios in RA are also altered. In BXD2 mice, a model of erosive arthritis mediated by excessive production of autoantibodies, the Tfh/Tfr and B/Tfr ratios in the spleen are significantly higher than those in wild-type (WT) mice (84). In addition, the Tfh/Tfr ratio is increased in the peripheral blood of patients with RA compared to that in HCs and is positively correlated with the DAS28 index (22). Indeed, Wang et al. (76) found that the circulating Tfr/Tfh ratio is inversely correlated with serum levels of CRP, erythrocyte sedimentation rate (ESR), RF, anti-CCP, IgG, and the DAS28 index. The Tfr/Tfh ratio is low in patients with RA, although the absolute numbers of Tfh cells and Tfr cells are higher, which suggests greater expansion of Tfh cells than Tfr cells in RA (76). Thus, at least part of the pathological mechanism of RA seems to involve aberrant expansion of Tfh cells and insufficient suppression by Tfr cells. Concordantly, a recent study showed that patients with RA in stable remission have a significantly higher blood Tfr/Tfh ratio than those with active RA and HCs (23).

The clinical significance of circulating Tfr cells, Tfh cells, and the Tfr/Tfh ratio needs to be confirmed because they may be markers for RA diagnosis and disease severity assessment. Heterogeneity of patients among studies may explain the controversy over the role of Tfr cells in RA. For example, patients in the study of Liu et al. (23) received no glucocorticoid and/or immunosuppressive drug within 1 month. In the study of Romão et al. (74), patients were treated with methotrexate, with or without other csDMARDs and/or glucocorticoids. It has been reported that methotrexate and DMARDs can significantly alter the number of circulating Tfr cells (76, 83). The controversy might be due to the use of different treatment regimens in patients (11). The definition of remission in RA is also inconsistent in these studies. The active disease group in Romão et al. (74) involves long-standing RA patients with DAS28 > 3.2. But the active group is defined as any joint with active disease or any sign of systemic disease by Liu et al. (23). In addition, the development of RA is dynamic, and we speculate that the number of circulating Tfr cells differs according to disease duration. As stated by Deng et al. (11), Tfr cells and Tfh cells can be induced and expanded by self-antigen stimulation. However, the expansion of Tfh cells is greater than that of Tfr cells, probably leading to an excessive GC response in RA (11). We speculate that the increased number of Tfr cells in RA can be explained as a reaction to the excessive autoimmune response. On this assumption, the number of circulating Tfr cells is more likely to increase significantly in active RA patients than in patients with inactive RA. Hence, the Tfr/Tfh ratio has greater clinical significance than the absolute number of Tfr cells or Tfh cells. In addition, the different sample sizes of previous studies may also explain the diversity of their conclusions. Further studies need to take these factors into consideration and control sample heterogeneity.

Compared to the frequency of Tfr cells, much less is known regarding the functions of Tfr cells in RA. In Liu et al. (23), Tfr cells from peripheral blood of patients with RA in stable remission and HCs were cocultured with Tfh cells and B cells, respectively. They found that the suppressive effects of Tfr cells in RA with stable remission were enhanced (23). Moreover, circulating Tfr cells from patients with MS have a reduced suppressive effect compared to the equivalent cells from HCs (21). Similarly, Tfr cells in patients with RA might be functionally deficient. Notably, FOXP3^+^ Tregs suppress ectopic lymphoid structure (ELS) generation in chronic inflammation (85). ELSs develop in the synovium of a minority of patients with RA (86), which contributes to RA by inducing local production of autoantibodies (87). Tfr cells have been found in the ELS in minor salivary glands of patients with SS, and the blood Tfr/Tfh ratio is associated with ELS formation in the minor salivary glands (78). Given that Tfr cells are also FOXP3^+^, whether they suppress the generation of ELSs and the underlying mechanism warrant further study.

It is important to note that chronic inflammation in RA is associated with many other cell types, for example, B cells, monocytes, fibroblasts, and peripheral helper T cells (73). Rao et al. (88) found a population of PD-1^hi^ CXCR5^−^ CD4^+^ peripheral helper T cells (Tph) capable of promoting B-cell responses and antibody production through IL-21 and CXCL13 in the RA synovium. The frequency of Tph cells is significantly higher in seropositive RA synovial fluid than that in seropositive RA patients (88). The reduction of disease activity parallels the reduction in the frequency of Tph cells in RA after medication (88). Notably, Th17 cells, newly defined T effector cells, induce tissue inflammation and organ-specific autoimmunity by producing proinflammatory cytokines such as IL-17, IL-6, IL-21, and IL-22; all of these cytokines are involved in the pathology of RA (89, 90). The imbalance between Tregs and Th17 cells may be involved in autoimmunity (91). A recent study based on a large number of samples from inflamed joints revealed the potential of sublining synovial fibroblasts as a therapeutic target in RA. Four synovial fibroblast subpopulations were identified based on their major histocompatibility complex (MHC) II expression and cytokine production. Different synovial cell types seem to drive different inflammatory pathways in patients with RA and those with osteoarthritis (73).

The mechanism underlying the altered number of Tfr cells and Tfh cells in RA warrants further investigation. Given the issues with obtaining Tfh cells and Tfr cells from secondary lymphoid organs, most findings are based on circulating Tfr cells and Tfh cells. Whether similar changes in the number of Tfr cells and Tfh cells occur in the GCs of secondary lymphoid organs and synovial tissue of RA patients is not clear. Moreover, whether Tfh cells also regulate Tfr cells should be studied. Significantly, some seronegative patients with RA lack RF and anti-CCP in serum. This raises the question of what roles Tfr cells and Tfh cells play in seronegative patients with RA.

## Potential Targets in TFR Cells

### Modified Differentiation of TFR Cells via the mTOR Pathway

The mTOR pathway is a key regulator of the development of Tfr cells (40, 62). mTOR is a serine/threonine kinase that integrates various messages to dictate gene transcription and translation, as well as apoptosis, autophagy, and proliferation (92). There are two distinct complexes of mTOR, mTOR complex 1 (mTORC1) and mTOR complex 2 (mTORC2) (92). Tfr cells exhibit elevated mTORC1 signaling compared to Tregs after antigen stimulation (62). Current studies indicate that the mTOR pathway can regulate the development of Tfr cells. For instance, Roquin inhibits the PI3K-mTOR pathway at several levels, consequently inhibiting the conversion of Tregs into Tfr cells (40). The deletion of Rptor, an essential component of mTORC1, leads to impaired differentiation of Tfr cells in mice (62). This impairment is reflected in reduced levels of CXCR5, glucocorticoid-induced TNF receptor family-related protein (GITR), and CTLA4 in Tfr cells (62). Mechanistically, mTORC1 induces the expression of the transcription factor TCF1 by phosphorylating STAT3, which promotes expression of Bcl-6 in Tregs and upregulates CXCR5 in Tfr cells (62). These findings shed light on the importance of the mTORC1-pSTAT3-TCF-1 axis in the differentiation of Tfr cells. Moreover, mTOR pathway is also required for the function of Tfr cells. The expression of components of the mTORC1 pathway in B cells was reduced during suppression by Tfr cells (65). Differentiated Tfr cells showed attenuated function in suppressing the expansion of B cells when treated with rapamycin (a mTORC1 inhibitor) (62).

Evidences suggest that the mTOR pathway is a potential target against RA and other autoimmune diseases by restoring functional and numeric Tfr disorders. Baicalin, a natural compound isolated from Chinese herb, restores the balance of Tfh and Tfr cells by inhibiting the mTOR signaling pathway, leading to amelioration in lupus nephritis (93). The combination of an mTOR inhibitor and vitamin D3 prevented bone destruction in RA, as the PI3K/Akt/mTOR pathway is critical for osteoclast differentiation and survival (94). Rapamycin suppresses the erosion of fibroblast-like synoviocytes in the synovial tissue of patients with RA (95). As mentioned above, the mTOR pathway is involved in multiple biological processes in addition to regulating Tfr cell development; therefore, targeting mTOR to regulate Tfr cells may induce side effects. To achieve precise regulation of Tfr cells via the mTOR pathway, further studies need to clarify the underlying mechanisms. Identification of downstream targets in the mTOR signaling pathway or combinations of multiple targets might avoid unwanted effects and achieve a balanced Tfr/Tfh ratio in RA.

### Regulation of the Differentiation and Function of Tfr Cells by Epigenetic Modification

MicroRNAs (miRNA) are regulatory small non-coding RNAs, which play an important role in gene expression, particularly at the posttranscriptional level. miRNAs regulate the immune response by suppressing the expression of key immune-associated genes (96, 97). In genome-wide expression analyses, miR-146a is highly expressed in human Tfh cells, and miR-146a deficiency leads to the accumulation of Tfh cells and GC B cells. Mechanistically, miR-146a suppresses the responses of Tfh cells and GC by repressing ICOS signaling (41). Moreover, the miR-17–92 cluster acts as a crucial regulator of Tfh cells by promoting the differentiation of Tfh cells. In mice with miR-17–92-deficient T cells, the number of splenic Tfh cells was significant decreased (98). Overexpression of the miR-17–92 cluster in T cells in lymphocytes leads to autoimmunity in mice (99). However, the role of miR-17–92 in the development of Tfr cells is unclear. Although overexpression of miR-17–92 in CD4^+^ T cells results in an increased frequency of Tfr cells (98), it has been proposed that miR-17–92 suppresses Tfr cell generation in mice with chronic graft-versus-host disease (100). A possible mechanism by which miR-17–92 regulates Tfr cells is targeting Pten (a PI3K inhibitor) in Tregs, thereby enhancing PI3K-Akt-mTOR signaling (40). Other miRNAs implicated in Tfr cell differentiation are summarized in Table 1. Given the impact of miRNAs in Tfr and Tfh cell development, it is likely that miRNAs could be a regulator in Tfr/Tfh balancing during RA development. Notably, miRNA-based treatments are effective in several autoimmune diseases. For example, in a mouse model of lupus-like chronic graft-versus-host disease, inhibitors of miR-17 alleviate clinical manifestations (100). Thus, it is likely that miRNA clusters that promote Tfr cell generation and inhibitors of miRNA clusters that negatively regulate Tfh cells could restore the Tfr/Tfh balance in RA patients with a decreased Tfr/Tfh ratio.

Epigenetic modification by histone deacetylase (HDAC) is closely related to chronic inflammation and autoimmunity (101, 102). HDAC regulates cell differentiation or death at the transcriptional level (103) and possesses anticancer activity (104). Moreover, HDAC9 deficiency leads to decreased autoantibody production and Bcl-6 expression in MRL/lpr mice (101). In models of colitis, strategies to reduce HDAC9 expression enhance the function of Tregs and prevent colitis (105). Thus, we speculate that HDAC inhibitor might also regulate the function and/or generation of Tfr cells at the transcriptional level because the phenotype and function of Tfr cells are similar to those of Tregs. HDAC inhibitors (HDACi) suppress the expression of IL-4, interferon (IFN)-γ, TNF-R, and IL-7R and are used in organ or bone marrow transplantation to induce immune tolerance (106). Further studies are needed to address whether HDACi regulates the expression of IL-21, Bcl-6, and other molecules involved in Tfr cell development.

### Tfr Cells and the Gut Microbiota

The microbiota have been investigated intensively in recent years given its profound impact on immunity. The microbial communities, their metabolites, and components may act as pathogen-associated molecular patterns (PAMPs), which are detected by pattern recognition receptor and activate antigen-presenting cells to initiate an immune response (107, 108). However, little is known about the mechanism by which the gut microbiota modulate autoimmune diseases.

The gut microbiota have both anti-inflammatory and proinflammatory functions. Segmented filamentous bacteria (SFB) promote the generation of Th17 cells in the gut and induce autoimmune arthritis in K/BxN mice (109). They also induce PP Tfh cell differentiation and migration into systemic sites, leading to autoimmune arthritis (110). By contrast, capsular polysaccharide A of *Bacteroides fragilis* induces the differentiation of CD4^+^ T cells into Tregs, thereby suppressing inflammation (111, 112). Moreover, butyrate, a common intestinal metabolite, promotes Treg differentiation (113). Tfr cells are derived from Tregs, and the gut microbiota might affect Tfr cell differentiation by regulating Tregs. Notably, short chain fatty acid (SCFA) produced by the microbiota modulates the production of antimicrobial peptides, which regulate intestinal homeostasis by activating mTOR and STAT3 in intestinal epithelial cells (IECs) (114). As mentioned above, the mTORC pathway is involved in the differentiation of Tfr cells. Therefore, microbial metabolites might regulate the Tfr/Tfh balance to induce immune tolerance through the mTORC pathway. Moreover, Tfr cells also have an impact on gut microbiota. Foxp3^+^ T cells facilitate diversification of the gut microbiota, but this is suppressed by selective inactivation of Bcl-6 (115). Because Foxp3^+^ T cells differentiate into Tfr cells in a Bcl-6-dependent manner, Tfr cells likely contribute to maintenance of the diversity of the gut microbiota.

Importantly, regulation of the gut microbiota can alleviate RA. In a mouse model, *Lactobacillus* reduces pannus formation, synovial infiltration, and bone destruction (116). In a clinical trial, patients with RA who consumed *Lactobacillus casei* for 8 weeks showed a significant decrease in disease activity score, along with reduced serum levels of proinflammatory cytokines such as tumor necrosis factor-α, IL-6, and IL-12 (117). As mentioned above, these cytokines are involved in the development of Tfr cells.

Altogether, cross talk between Tfr cells and gut microbes such as SFB, *B. fragilis*, and *Lactobacillus* may be involved in the development of RA, which warrants further exploration.

### The IL-2–IL-21 Axis in the Tfh and Tfr Cell Balance

IL-2, a pleiotropic cytokine mainly produced by CD4^+^ T cells after antigen stimulation, has a function in maintaining the generation and function of Tregs (118, 119). Tregs suppress GC B-cell responses and autoantibody production (120). And Foxp3 is required for the development of Tregs (121, 122). IL-2 upregulates Foxp3 expression in Tregs via Jak/STAT5 signaling and thereby promotes Treg-mediated immune suppression *in vivo* (123, 124). Tregs are sensitive to IL-2, as indicated by their higher expression of *Il-2Ra* than other Teff cells (125). It is generally believed that low-dose IL-2 selectively activates and promotes the generation of Tregs (126). Interestingly, the number of Tregs in the thymus is increased in *Il-2Ra*^−/−^Tg mice, but their suppressive effect is decreased (63). In contrast, peripheral Tregs have an unchanged suppressive effect *in vitro* and activated Th1-like phenotype (63). Importantly, low-dose IL-2 inhibits the generation of human Th17 in SLE and pSS (127–129). Related to promoting Treg generation and/or suppressing Th17 differentiation, low-dose IL-2 has been known as a strategy to ameliorate various immune-mediated disorders (127, 130).

IL-2 inhibits the differentiation of Tfh cells *in vivo* (131). The number of Tfh cells is positively correlated with the intensity of the humoral response (132). Mechanically, IL-2 represses Bcl-6 expression via the STAT5 pathway, which precludes the differentiation of Tfh cells and so controls the GC reaction (133, 134). Bcl-6 is critical for Tfr cell differentiation (16). Given the repressed expression of Bcl-6 by IL-2 in Tfh cells, low-dose IL-2 might inhibit Tfr cell generation. Botta et al. (39) showed that high concentrations of IL-2 at the peak of the infection preclude Treg development into Tfr cells by promoting Blimp-1, an essential suppressor in Bcl-6 expression. In addition, Bcl-6 and CXCR5 are upregulated, which is required for development into Tfr cells, in Foxp3^+^ cells cultured in the presence of a low level of IL-2. Interestingly, Tfr cells with different origin showed different responsiveness to IL-2. PP Tfr cells have a robust downregulation of *Il-2Ra* and remained largely unresponsive to IL-2, while the expression of *Il-2Ra* on Tfr cells of pLN origin is high, suggesting that IL-2 signaling pathway is operative in these cells (28).

Although IL-2 negatively regulated Tfr cell differentiation, blocking TGF-β and IL-2 signaling disrupts Treg phenotype and function as well as their differentiation into Tfr cells. This leads to an increased number of Tfh cells and enhanced GC response (63). Thus, IL-2 plays an important role in the development of Tregs and Tfr cells. Disruption of the role of IL-2 in Tfr, Tfh, and Treg cells development might be involved in RA. The pathway by which IL-2 regulates Tfr cell differentiation and how IL-2 influences the function of Tfr cells in RA need to be defined.

IL-21, mainly produced by Tfh cells, modulates the GC reaction by counteracting the suppression of Treg and promoting the production of antibodies (135, 136). Blockade of IL-21 reduces disease severity in mice with RA, indicating that IL-21 is closely related to RA progression (137). In addition, IL-21 promotes Tfh cells but inhibits Tfr cell development in BXD2 mice (24). Sage et al. (65) found that high concentrations of IL-21 overcome Tfr cell-mediated suppression of B cells. Consistent with this, IL-21 reinforces the humoral immune response by inhibiting the proliferation of Tfr cells (38). Mechanically, IL-21 signaling has a negative effect on the expression of CD25 by upregulating Bcl-6, which reduces responsiveness to IL-2 and decreases the proliferation of CD25^+^ Tfr cells (38).

These findings suggest that IL-21 and IL-2 regulate Tfr development and function, and their balance is key for maintaining a normal Tfr/Tfh ratio. Indeed, impaired IL-2 production, a high IL-21 concentration, and an aberrant Tfr/Tfh ratio are typically found in patients with RA (22, 76, 138). In the CIA model, blockade of the IL-21 pathway ameliorates disease (137). In SLE patients, low-dose IL-2 treatment modulated CD4^+^ T-cell subsets and reduced disease activity (127). The mechanisms by which IL-21 and IL-2 regulate inflammation via Tfr cells and Tfh cells remain to be determined; such information could help prevent RA.

## Conclusions and Future Perspectives

Tfr cells are critical mediators of GC response with great therapeutic potential in RA, although their role in autoimmune diseases is controversial. Regulation of Tfr cells requires various regulators such as mTOR signaling, HDACi, microRNA, cytokines, and the gut microbiota. These regulators are closely linked and largely unclear. For instance, miR-17–92 targets PTEN, thus enhancing PI3K-Akt-mTOR signaling (40). SCFAs are important HDACi (113). Indeed, RA is a complex autoimmune disease with multiple regulatory mechanisms. The therapy only by Tfr cell regulation in RA might have limitations. Combinations of multiple strategies could pave the way for the development of immunotherapies for RA. It is important to systematically assess the correlation between the number of circulating Tfr cells and markers of the diagnosis, severity, treatment efficacy, and prognosis of RA. Moreover, the relationship among circulating Tfr cells, tissue Tfr cells, and PP Tfr cells should be clarified. We believe that Tfr cells represent an attractive therapeutic target in RA and warrant further mechanistic studies and clinical trials.

## Author Contributions

TD drafted the manuscript, prepared illustrations, and discussed the content with the other authors. CW conceived the topic and revised the manuscript. HN intellectually revised the manuscript. XZ, CG, and XL also critically revised the manuscript for intellectual content. All of the authors approved the manuscript for publication.

### Conflict of Interest

The authors declare that the research was conducted in the absence of any commercial or financial relationships that could be construed as a potential conflict of interest.
